# Classification of Protein Kinases on the Basis of Both Kinase and Non-Kinase Regions

**DOI:** 10.1371/journal.pone.0012460

**Published:** 2010-09-15

**Authors:** Juliette Martin, Krishanpal Anamika, Narayanaswamy Srinivasan

**Affiliations:** 1 Molecular Biophysics Unit, Indian Institute of Science, Bangalore, India; 2 INRA UR1077, Unité Mathématique Informatique et Génome, Jouy-en-Josas, France; University of California Riverside, United States of America

## Abstract

**Background:**

Protein phosphorylation is a generic way to regulate signal transduction pathways in all kingdoms of life. In many organisms, it is achieved by the large family of Ser/Thr/Tyr protein kinases which are traditionally classified into groups and subfamilies on the basis of the amino acid sequence of their catalytic domains. Many protein kinases are multi-domain in nature but the diversity of the accessory domains and their organization are usually not taken into account while classifying kinases into groups or subfamilies.

**Methodology:**

Here, we present an approach which considers amino acid sequences of complete gene products, in order to suggest refinements in sets of pre-classified sequences. The strategy is based on alignment-free similarity scores and iterative Area Under the Curve (AUC) computation. Similarity scores are computed by detecting common patterns between two sequences and scoring them using a substitution matrix, with a consistent normalization scheme. This allows us to handle full-length sequences, and implicitly takes into account domain diversity and domain shuffling. We quantitatively validate our approach on a subset of 212 human protein kinases. We then employ it on the complete repertoire of human protein kinases and suggest few qualitative refinements in the subfamily assignment stored in the KinG database, which is based on catalytic domains only. Based on our new measure, we delineate 37 cases of potential hybrid kinases: sequences for which classical classification based entirely on catalytic domains is inconsistent with the full-length similarity scores computed here, which implicitly consider multi-domain nature and regions outside the catalytic kinase domain. We also provide some examples of hybrid kinases of the protozoan parasite *Entamoeba histolytica*.

**Conclusions:**

The implicit consideration of multi-domain architectures is a valuable inclusion to complement other classification schemes. The proposed algorithm may also be employed to classify other families of enzymes with multi-domain architecture.

## Introduction

Kinases constitute a key class of enzymes responsible for the regulation of many biological phenomena. By covalently attaching a phosphate group to its target (phosphorylation) a kinase is able to regulate a particular biological reaction. Kinases are virtually involved in almost every signal transduction pathway occurring in a living cell. Although different types of biological molecules can be phosphorylated (proteins, nucleotides, lipids, etc), the largest group of kinases is protein kinases, which phosphorylate at Ser/Thr or Tyr residues. About 2% of proteins encoded in genomes of most of the eukaryotes are indeed Ser/Thr/Tyr kinases [1]. They are involved, for example, in cell cycle control, embryonic development, as well as in cancer pathways, and thus constitute popular drug targets [2]–[4].

Genome sequencing projects generate data at a rate that makes it impossible to conduct biological experiments to characterize the function of every protein encoded in a genome. In this context, several groups, including ours, have developed bioinformatic approaches to identify and analyze the repertoire of protein kinases (kinomes) in complete genomes [5]–[22]. Our KinG database [1] (http://hodgkin.mbu.iisc.ernet.in/~king/) currently stores the analysis made from 488 genomes: 54 eukaryotes, 49 archaebacteria, 259 eubacteria, and 126 viruses. Ser/Thr/Tyr kinases form a very large protein family. They are divided into many subfamilies which usually correspond to different substrate specificities and mode of activation. Classification of protein kinases encoded in a genome into various subfamilies is extremely valuable to gain further insight into their detailed biological function. However the available classification approaches as used, for example, in the construction of KinG and Kinomer databases [23], use the amino acid sequences of catalytic kinase domain only and ignores the sequence of the regions outside the catalytic domain.

Several classification schemes for kinases have been proposed in the literature. In their path paving work, Hanks and Hunter performed conservation and phylogeny analysis of the catalytic domains of eukaryotic protein kinases [24], [25]. It enabled them to reveal conserved features of the catalytic domain; furthermore, protein kinases with similar mode of regulation clustered together in the resulting phylogenetic tree [24]. In a subsequent paper, they exhaustively applied the phylogenetic analysis to all eukaryotic protein kinases whose sequence was available at the time of their study, i.e., 243 sequences. Based on the resulting phylogenetic trees, they proposed a classification scheme consisting in 5 major groups, divided into 55 subfamilies, with related substrate specificity and mode of regulation [25]. This classification scheme which is entirely dependent on the amino acid sequences of catalytic domains only, is currently used to describe the sequences stored in our KinG database. The Hanks and Hunter classification has then been extended by different groups. An extended version of Hanks and Hunter classification has been developed for the Protein Kinase Resource [26], [27] (http://www.nih.go.jp/mirror/Kinases/). It is composed of three levels: 9 groups, 81 families and 238 subfamilies. Another extension of Hanks and Hunter classification was developed by Manning and coworkers for the KinBase database (http://www.kinase.com/kinbase). Families were obtained by sequence comparison of the catalytic domains, aided by knowledge of sequence similarity and domain structure outside of the catalytic domains, known biological functions, and gross similarity in biological functions of kinases across organisms [7], [28]. It currently consists of 10 groups divided into 256 families. Some of the 256 families are organism-specific. This classification has been subsequently used by Miranda and Barton, who proposed a multilevel classification procedure based on Hidden Markov Model (HMM) profiles of catalytic domains for sequence classification [29]. This classification scheme has been used to describe the sequences stored in the Kinomer database [23]. The Kinase Sequence Database (http://sequoia.ucsf.edu/ksd/), contains 7128 protein kinases from 948 organisms, classified into 287 families [30]. The clustering into subfamilies is achieved by a recursive algorithm combining BLAST and profile-based searches. An all-against-all BLAST search is first carried out to produce a set of pairwise scores. The top-scoring pair is then used to generate a HMM profile and the sequences matching that profile are integrated into the family and removed from the data set. This procedure is iterated until exhaustion of the pool of sequences or on reaching the lower limit of BLAST score. The KSD classification scheme relies only on the catalytic domain sequences and it does not consider non-kinase regions in multi-domain kinases. A specific classification for plants is used in PlantsP (http://plantsp.genomics.purdue.edu/), a database dedicated to plant protein kinases and phosphatases [31], [32]. In this approach protein kinase sequences were clustered using scores provided by full-length sequence comparison using BLAST and maximal linkage clustering. This resulted in a classification with three levels: 5 classes divided into 27 groups, divided further into 44 families. This classification, however, is specifically designed for plant protein kinases. A comprehensive classification of all kinases, not restricted to protein kinases, was carried out by Cheek and colleagues [33], [34]. They built HMM profiles corresponding to Pfam and COG families of catalytic kinase domains [35]–[37] and used them to retrieve all putative kinases from a non-redundant database. Evolutionary links between profiles were then detected using PSI-BLAST and related families were merged, resulting in a final classification into 11 groups, divided into 25 families and encompassing 59,402 sequences. In the resulting classification, all protein kinases belong to the same family of 22,074 sequences, other families separating kinases for other substrates. Finally, structural information can also be incorporated into the classification. Scheeff and Bourne thus combined sequence alignment and structural features to perform Bayesian phylogenetic inference, yielding a phylogenetic tree encompassing 31 protein kinases [38]. Recently, Jabobs et al classified 426 structures corresponding to 71 distinct human protein kinases, based on the conformations of two structural elements. The resulting clusters were in agreement with inhibitor specificity of the kinases [39]. Unfortunately, structural information is available only for a small fraction of protein kinases, making these approaches not suitable for whole kinome analysis. It thus appears that the main classification schemes currently in use for full kinome analysis are based on or derived from the Hanks and Hunter pioneering classification that relied solely on the catalytic domain sequences. They do not automatically make use of information on accessory domains that are found tethered to the catalytic kinase domain [8], [15], [16].

In this paper, we present a strategy to detect outliers in existing kinase classification. Our strategy is based on an alignment-free scoring scheme, which implicitly considers domains tethered to catalytic kinase domain. Similarity between sequences is assessed by the presence of common short amino acid patterns. This is an extension to the measure introduced by Kelil et al [40], with a consistent normalization scheme. These similarities are then used as input to an iterative procedure to detect outliers, i.e., proteins with inconsistent classification. We report the result of the outlier detection on the human kinome and the kinome of *Entamoeba histolytica*.

## Results and Discussion

We first present some notable features of protein kinases that explain the difficulties of kinase classification and led us to develop this approach. We then use a small data set of well characterized kinases and compare different types of distances (alignment-based and alignment-free) both on full-length sequences and catalytic domains. The correlation between them and their ability to form homogeneous clusters is assessed. We finally report some potentially interesting cases that emerge from the analysis of the entire human kinome using alignment-free distances, and a brief analysis of the *Entamoeba histolytica* kinome.

### Specific features of protein kinases

#### Many protein kinases are multi-domain proteins

The functional catalytic core of all Ser/Thr/Tyr kinases is a domain of approximately 250–270 amino-acids with a common three-dimensional fold [41]. In many eukaryotic protein kinases, as well as in a number of prokaryotic protein kinases, this catalytic domain is tethered to one or more non-kinase domains that are responsible for regulation, substrate specificity, scaffolding, etc [42]. Globally, 64% of the kinases analyzed in KinG database have at least one accessory domain tethered to the catalytic kinase domain. If we restrict to the human kinome, 330 kinases out of about 550 are multi-domain proteins. There is also a wide variety of different protein architectures, as listed in Table 1, with 65 distinct domain architectures seen in the validation data set of 212 human kinase sequences. The presence of accessory domains is thus a non-negligible factor, that should be taken into account for the kinase classification. In an early attempt, we have tested the use of distance measures based on domain architectures, with moderate success (see Text S1). Indeed, an inherent limitation of such distances is that they rely heavily on the domain detection step. Difficulties in this step are discussed in the next paragraph.

**Table 1:** Composition of the validation data set.

Hanks and Hunter classification group	Nb(prot)^1^	Nb(archi)^2^
Agc	37	17*
camk	35	7*
Ck1	6	1*
cmgc	38	1*
mekk_ste11	2	2*
mek_ste7	3	1*
mlk	3	1
nima	4	2*
pak	4	1
plantrk	3	3*
polo	1	1
Ptk	51	31*
Raf	2	1
tgfb	10	4*
translationk	2	2*
wee1	2	1*
unclassified	9	1*
Total	212	65

1 : number of proteins,

2 : number of distinct domain architectures,

*: contains the basic architecture made of a single catalytic domain.

#### Domain detection can be difficult in some cases

It is not uncommon that significant proportion of a long protein sequence is unassigned in terms of domains. For example, a very long protein kinase of several thousands of residues can be described by a catalytic domain that only spans a small portion of it and much of the rest of the sequence might remain uncharacterized by any standard domain family. In the KinG database, the coverage of sequences by Pfam domains, on a per-residue basis, ranges from 6 to 100%. Low coverage typically refers to very long protein kinases with a catalytic region as the only detectable domain. Conversely, total coverage is often found in short proteins that match catalytic domain profile almost on their whole length. For human kinases, the coverage is between 11 and 97%, with an average of 58%.

The average coverage of kinomes stored in KinG by Pfam domains is highly variable across different organisms. For example, on a per-residue basis, it is equal to 21% for *Prochlorococcus marinus*, 43% for *Plasmodium falciparum*, and 80% for *Phycomyces blackesleeanus*. It means that, depending on the organism, 20 to 80% of sequence length in kinases have no detectable match with Pfam domain families. This reflects both on the variety of kinome organizations, and the fact that domain detection is impaired on highly divergent sequences such as those found in *Plasmodium falciparum*. In the cases of proteins with two kinase domains in a gene product, it is not trivial to consider subfamilies of both the kinase domains in the classification.

#### Domain shuffling occurs in protein kinases

Another feature of multi-domain protein kinases that complicates the classification is that they display domain rearrangement, i.e., domain A is followed by domain B in a kinase, and domain B is followed by domain A in another kinase. Some examples of domain shuffling seen in protein kinases are listed in Table 2. It can be seen that different domains can be tethered on both sides of the catalytic domains. In some cases, alternate domain architectures are seen within the same organisms.

**Table 2:** Examples of domain swapping in protein kinases.

Alternate domain architectures^1^	Organisms in which the 2 architectures are seen^2^
Ank Pkinase/Pkinase Ank	-
Pkinase Death/Death Pkinase	*Bos taurus*, *Branchiostoma floridae*, *Gallus gallus*,
	*Snipe's laevis*, *Xenopus tropicalis*
FHA Pkinase/Pkinase FHA	*Roseiflexus sp.* (strain RS-1),
	*Saccharomyces cerevisiae* (strain AWRI1631)
	*Trichodesmium erythraeum* (strain IMS101)
PP2C Pkinase/Pkinase PP2C	*Arabidopsis thaliana*, *Micromonas pusilla CCMP1545*,
	*Micromonas sp. RCC299*, *Oryza sativa subsp. japonica*,
	*Ostreococcus tauri*, *Vitis vinifera*
SH2 Pkinase/Pkinase SH2	-
UBA Pkinase/Pkinase UBA	-
Pkinase PH/PH Pkinase	*Entamoeba dispar SAW760*, *Trypanosoma cruzi*

1 : domain architectures are searched against PFAM 24.0,

2 : for each architecture, corresponding sequences are retrieved from PFAM 24.0, and the origin organism is considered. Abbreviations used: Pkinase: protein kinase catalytic domain, Ank: ankyrin repeat, Death: death domain, FHA: Forkhead-associated domain, PP2C: protein phosphatase 2C, SH2: src homology 2 domain, UBA: ubiquitin associated domain, PH: pleckstrin homology domain.

### Comparison between different distances

The classification of protein kinases is classically based on the comparison of amino acid sequences of catalytic domain regions alone. Given the (i) multi-domain nature of many protein kinases, (ii) difficulty of domain assignment, and (iii) existence of domain shuffling, we wished to propose a protocol to refine classification that would:

- take into account full-length sequences,
- be insensitive to domain detection inaccuracies by avoiding the step of domain detection,
- take into account domain shuffling.

These are achieved by Local Matching Score (LMS) measure, that satisfies these three conditions.

Here, we compare the distances derived from LMS with alignment-based distances: distance based on percentage identity between catalytic domains ( = identity distance), distance based on BLOSUM scores between catalytic domains ( = BLOSUM catalytic distance), and distance based on BLOSUM scores between full-length sequences ( = BLOSUM full distance). For comparison purpose, the LMS distances are computed for catalytic domains ( = LMS catalytic distance) and full-length sequences ( = LMS full distance). All distances are normalized between 0 and 1. Computation of distances are detailed in the Materials and Methods section. The validation data set consists of 212 human kinases with their classification well established.

#### Selected examples

**Figure 1:**
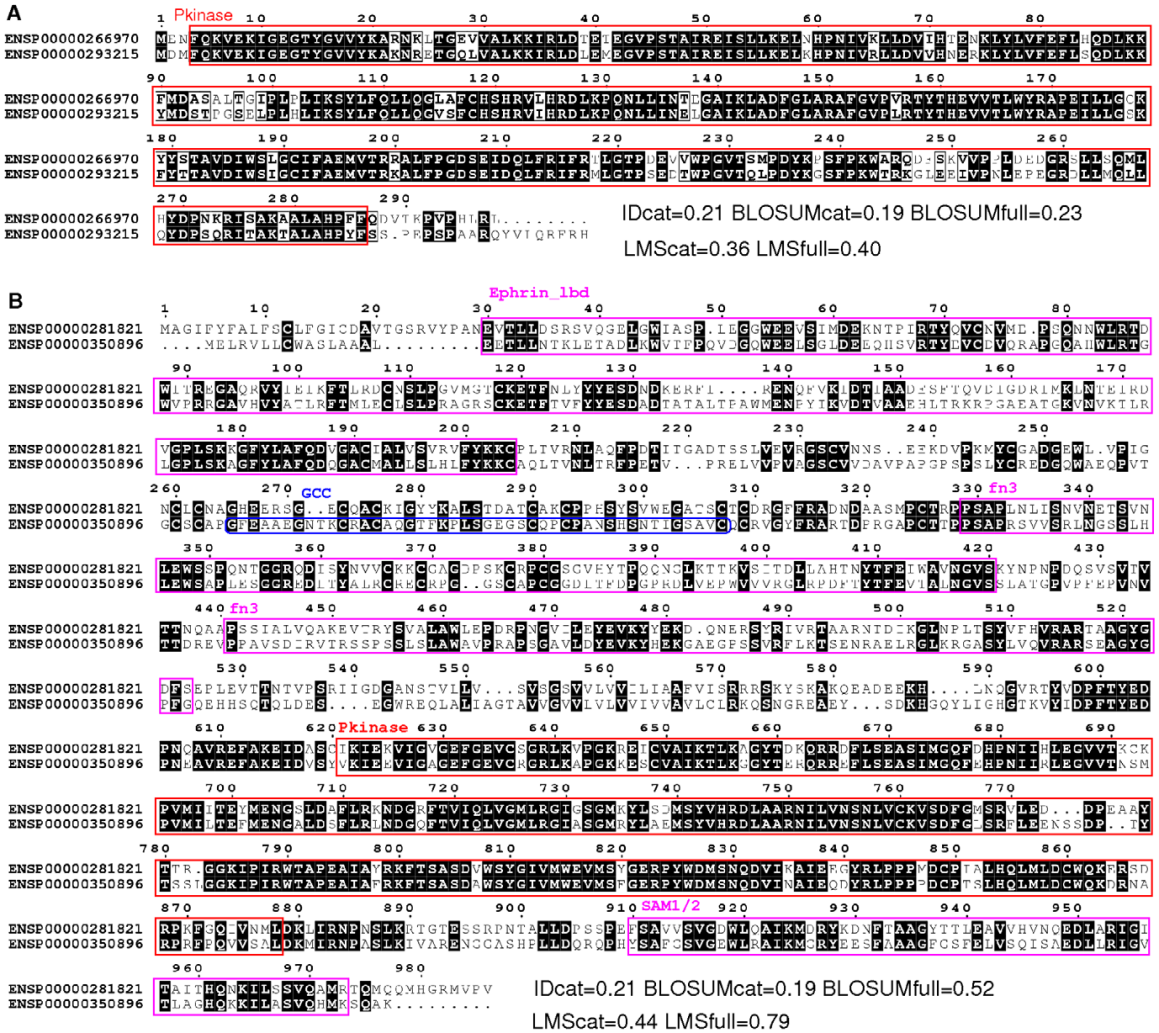
Sequence alignment of selected protein pairs. A: Sequences ENSP00000266970 and ENSP00000293215. B: Sequences ENSP00000281821 and ENSP00000350896. Identities are indicated by black background. Pfam domains are indicated by colored boxes: red = catalytic domains, magenta = domains detected in both proteins, blue = domain detected in only one protein. Abbreviations used: Ephrin_lbd = Ephrin receptor ligand binding domain, GCC = GCC2 and GCC3 domain, fn3 = fibronectin type III domain, Pkinase = protein kinase domain, SAM = sterile alpha motif domain (type SAM_1 is detected in ENSP00000350896 and type SAM_2 is detected in ENSP00000281821). Global sequence alignment is obtained using the Needleman-Wunsch algorithm. For each pair of sequences, the different distances are indicated at the bottom of the alignment. Image generated using ESPript software [52].

Figure 1 illustrates the difference between different types of distances on two selected pairs of human kinases Proteins. ENSP00000266970 and ENSP00000293215, aligned on Figure 1A do not possess any accessory domains or unassigned regions. Hence, distances computed from catalytic domains and full-length sequences give consistent results: about 0.2 using BLOSUM distances and 0.4 using LMS distances. On the contrary, proteins ENSP00000281821 and ENSP00000350896, shown in Figure 1B are multi-domain kinases. Their catalytic domains share high sequence identity, but these proteins are less similar if we consider their full lengths. The divergence of the sequences even results in different domain assignment in these two kinases: domain GCC is detected in ENSP00000350896 but not in ENSP00000281821. Indeed, distances based on catalytic domains are smaller than full-length distances: 0.2 *versus* 0.5 with BLOSUM distances, and 0.4 *versus* 0.8 using LMS distances Note that whether a GCC domain is detected in sequence ENSP00000350896 or not has no influence on full-length distance computation since the approach does not rely on domain definition. For the same reason, they allow to take into account the similarity in linker regions: in this example, the regions upstream the catalytic domains.

#### Global comparisons

**Figure 2:**
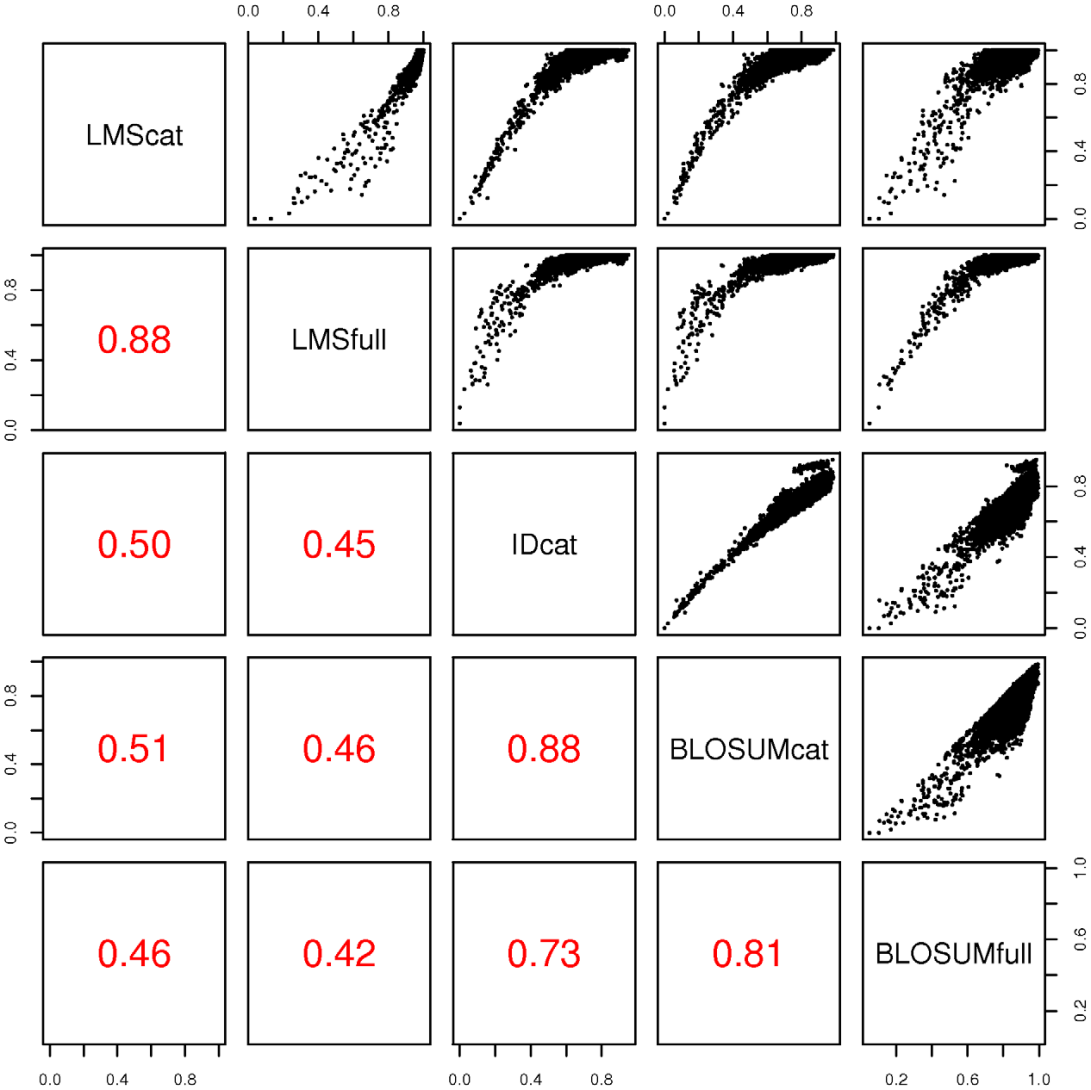
Comparison between the different distances computed between protein sequences of the validation data set. LMScat: LMS distances between catalytic domains, LMSfull: LMS distances between full-length sequences, IDcat: identity distances between catalytic domains, BLOSUMcat: BLOSUM distances between catalytic domains, BLOSUMfull: BLOSUM distances between full-length sequences. The lower panel reports the Spearman rank correlation coefficients between different distances.

Figure 2 presents the correlation between different distances computed from the validation data set. All types of distance are positively correlated, with Spearman correlation coefficients ranging from 0.45 to 0.88. As expected, sequence alignment-based, i.e., identity distances and BLOSUM distances show very high correlation, with coefficients between 0.73 and 0.88. These distances are all computed after sequence alignment, the difference between them being the way the alignments are scored, they are thus expected to give similar results. By contrast, LMS distances show lower correlation with alignment-based distances, with correlation coefficients between 0.42 and 0.50. These distances are based on the detection of common patterns between sequences without alignment, and are thus likely to bring additional information. Interestingly, on the comparison between full-length LMS distances and identity distances, as well as the comparison between full-length LMS distances and identity distances, points are scattered in the region corresponding to low identity distance. This scattering indicates that some pairs of sequences can appear in close proximity according to identity distance between their catalytic sites, and have various similarity level based on LMS distances.

These results underline the importance of considering regions outside catalytic kinase domain in classification, as sequence alignment-based measures tend to overrate the similarity levels between two multi-domain kinases with high similarity in the conserved kinase region and poor or almost no similarity outside the catalytic region.

#### Ability to generate homogeneous clusters

**Figure 3:**
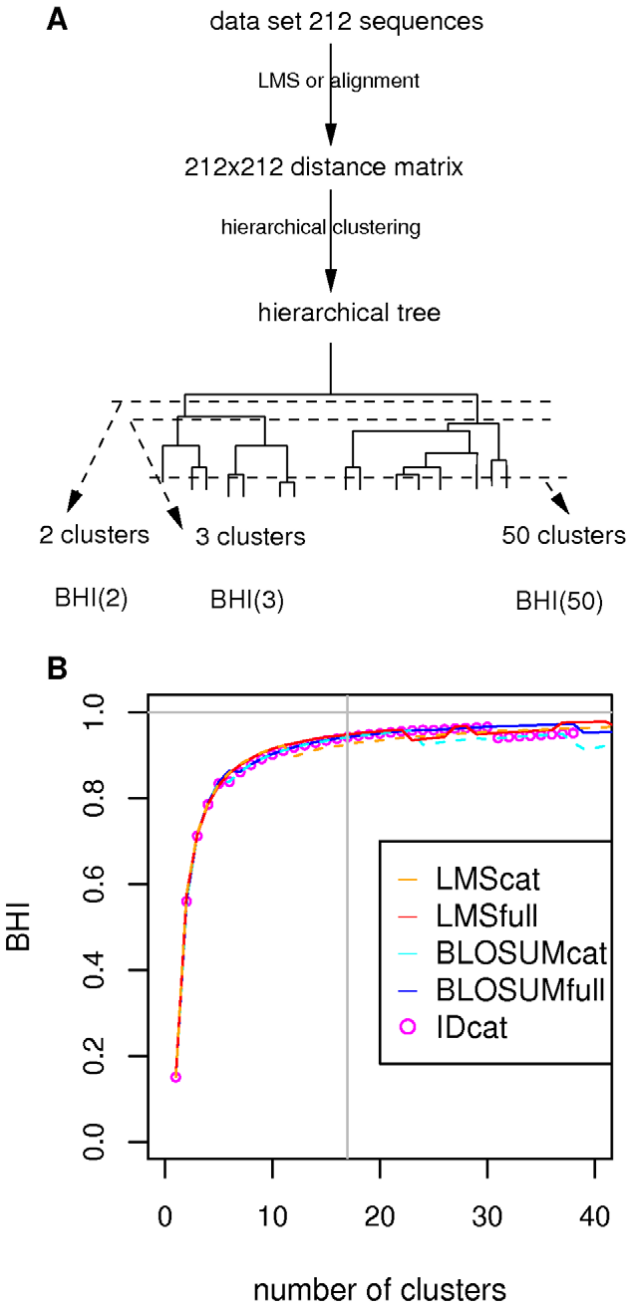
Assessment of different distances to detect homogeneous clusters in the validation data set. A: each distance matrix is used as input to hierarchical clustering; clusters are extracted from the resulting trees and assessed by the biological homogeneity index (BHI); B: evolution of BHI according to the number of clusters. LMScat: LMS distances computed from catalytic domains, LMSfull: LMS distances computed from full-length sequences, BLOSUMcat: Blosum distances computed from catalytic domains, BLOSUMfull: Blosum distances computed from full-length sequences, IDcat: identity distances computed from catalytic domains. Horizontal and vertical lines indicate respectively BHI = 1 and number of clusters equal to 17.

We assess the efficiency of the different types of distances to delineate pertinent biological groups in a data set of 212 human protein kinases. As explained in the Materials and Methods section, sequences of the validation data set are assigned into 17 broad families, derived from Hanks and Hunter classification, in agreement with Swissprot annotations. The ability of the distances to cluster these sequences in homogeneous groups is assessed using the procedure illustrated in Figure 3a. Briefly, distance matrices are used as the input to hierarchical clustering using Wards linkage; the resulting hierarchical trees are then cut at various depths, and the global quality of resulting clustering is measured by the Biological Homogeneity Index (BHI). A BHI close to 1 indicates homogeneous clusters with respect to the available annotations. In Figure 3b, we report the BHI as a function of the number of clusters, obtained using different distance matrices. It can be seen that the four types of distances yield clusters of high and roughly similar homogeneity. It should be noted that we do not expect a better performance of LMS distances over alignment-based distances when comparing catalytic domains only. Under such circumstances, sequence alignment is surely the most accurate way to evaluate the similarity between two sequences. The LMS scores are only an approximation of this similarity, based on common patterns. At 17 clusters, which is the number of different broad families in the data set, BHI is equal to 94.2% using identity and BLOSUM distances, 92.5% using the LMS catalytic distances and 94.8% using the LMS full distances.

### Outliers in the human kinome

Having shown that distances between full-length sequences are able to form homogeneous clusters on a small validation data set, we now employ them to detect outliers in the human kinome. We use the iterative procedure described in the Materials and Methods section, to detect sequences that are divergent compared to the core members of their group. These sequences constitute potential cases of uncertainty in classification. In some of these outliers, amino acid sequence of the catalytic region is most similar to a particular sub-family of kinases, but the regions outside the catalytic kinase region suggests similarity to another sub-family. We refer these cases as “hybrid kinases”.

Analysis of kinases in various organisms, ranging from prokaryotes to eukaryotes, revealed the presence of hybrid kinases [42]. For example, in the protozoan parasite *E. histolytica*, occurrence of few hybrid kinases has been reported [22]. Typical agc1 kinases are characterized by a single domain (the catalytic domain). However in four *E. histolytica* kinases with catalytic domains closely related to the agc1 subfamily, pleckstrin homology (PH) domain occurs before the kinase region in the primary structure. Such a domain architecture is typical of certain agc3 kinases, which act near plasma membrane. High similarity of the catalytic region to agc1 kinases suggest that some of the properties, such as substrate specificity and/or mode of regulation, of these hybrid kinases are inherited from agc1. However, given the existence of PH domain in these kinases, some other properties, like sub-cellular localization or involvement of phospholipids, may be inherited from agc3. One of the *E. histolytica* kinases shows good similarity to camk kinases in its catalytic region. However, atypical of camk kinases, this *E.histolytica* kinase has an endonuclease 5 domain following the kinase domain in the sequence [22]. This feature suggests that this kinase might interact with single-stranded or duplex DNA, while the substrates of this kinase would be closely-related to substrates of typical camks. Such unusual domain architectures in hybrid kinases may also suggest a role of these kinases in facilitating cross-talks between different signal transduction pathways. Some of the *E. histolytica* kinases show close similarity to cytoplasmic kinases in their catalytic kinase regions. However a transmembrane region is predicted in all these *E. histolytica* kinases. This suggests a hybrid nature in these kinases, inheriting the property of association with membrane, like a receptor kinase, and potentially with substrate specificities similar to that of a cytoplasmic kinase. Occurrence of kelch domain has been reported [22] in a few *E. histolytica* tyrosine kinases, which indicates roles of these kinases in regulatory and cytoskeletal function. In an extreme case of hybrid nature, both histidine kinase domain and Ser/Thr kinase domain have been observed in a cyanobacterial protein [15].

**Figure 4:**
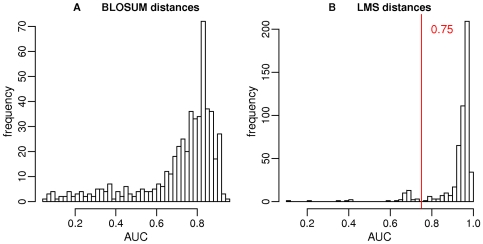
AUC distributions obtained on the human kinome. A: AUC obtained using the iterative procedure starting from BLOSUM full-length distances, B: AUC obtained using the iterative procedure starting from LMS full-length distances. The vertical red line indicates the cut-off for the detection of hybrid kinases.

The originality of our approach is to take into account the full-length sequences of all the kinases including hybrid kinases, such as those discussed above, and other multi-domain kinases. We have employed the procedure on the set of human kinases that were originally assigned, using the sequence of catalytic regions only, into one of the 55 standard subfamilies. During this classification, a number of kinases fell in the category of “unclassified kinases” as they did not show convincing level of similarity to any of 55 well-established subfamilies of protein kinases. These unclassified kinases were excluded in the present exercise. The final AUC distributions obtained with BLOSUM and LMS distances are presented in Figure 4. It can be seen that the histogram of AUC values obtained using BLOSUM distances exhibits continuous distribution, with no clear-cut threshold. On the contrary, we can easily set a threshold equal to 0.75 on the histogram obtained using LMS distances. Using this threshold, 38 sequences are detected as outliers, and proposed as putative hybrid kinases. In order to understand the hybrid nature of these outliers, we examined the closest sequences (in LMS distance) of each outlier and propose an annotation based on the neighbors. After a careful scrutiny, the final list of hybrid kinases comprises of 37 sequences. Summary information for these 37 cases is provided in Table S1, additional information is given in Table S2 and all AUC values are given in Table S3. In Table S1, we provide details about the catalytic domain similarity to known sub-families, available biological information and LMS similarity. In particular, we report the identity and sequence coverage percentage that was observed during our assignment to one of the Hanks and Hunter subfamilies using profiles of catalytic domains (see Materials and Methods section). Based on the LMS distances, and classification of closest neighbors, we suggest a classification which reflects hybrid nature of these kinases. In a number of cases, however, we are not able to suggest a hybrid annotation (question marks in Table S1), because the outlier has no close neighbors, or they are too diverse. Lack of closely-related kinases with good similarity in the catalytic region adds a further dimension of novelty to these kinases. We do not suggest that our classification as hybrid kinases should supplant currently existing classifications. We draw attention to the divergent similarity links that can exist among multi-domain kinases, when one restricts the comparison to catalytic domain or consider the full-length (multi-domain) sequences. Only experimental biochemical characterizations could provide ultimate confirmation. We discuss a few selected cases below.

#### ENSP00000303165

According to its catalytic domain, this kinase belongs to the agc1 subfamily (ATP-dependent and GTP-dependent protein kinases). It corresponds to Swissprot entry ST32A_HUMAN and is annotated as Ser/Thr protein kinase. There is evidence at transcript level for this protein, and no experimental data regarding the substrate or mode of regulation is available. Based on the LMS distance, this sequence is very similar to a sequence classified as agc_other. No known accessory domains were detected in this sequence, which is 358 amino acid long, although the number of residues outside the aligned catalytic region is nearly 100. Its catalytic domain, which is 259 residue long, displays distant similarity with the one of agc1: the match is characterized by 36% sequence identity with 99% coverage. There is thus a possibility that similarity outside the catalytic domain influences the classification of full length protein close to agc_other subfamily rather than agc1, which is assigned when one considers only the catalytic region.

#### ENSP00000155926

This kinase is classified as camk1, i.e., calcium/calmodulin regulated kinase, with distant similarity to camk1 catalytic domain profile: 30% identity and 94% coverage according to rps-blast search. This sequence corresponds to Swissprot entry TRIB2_HUMAN and is annotated as camk, belonging to the tribbles subfamily. There is evidence only at the transcript level for this protein. Like the previous case, there is a single catalytic domain detected in this sequence, covering 245 residues, for a total length of 343 residues leaving almost 100 residues outside the catalytic domain. Based on LMS distances the full-length sequence is classified as camk2 probably due to the sequence outside the catalytic region. Hence a hybrid camk1/camk2 status is assigned to this protein.

#### ENSP00000369030

This sequence belongs to the camk2 group, with low similarity in the catalytic domain: 30% identity over 94% coverage based on rps-blast. Again, no known accessory domain was detected in this sequence which is 483 residues long, with a catalytic domain of 263 residues. It corresponds to swissprot entry Q59GL9_HUMAN, for which there is evidence only at the transcript level, and appears close to an unclassified kinase using LMS distance, suggesting a hybrid camk2/unclassified status. One might expect the catalytic region of this protein recognizes substrates similar to that of a classical camk2, with regulatory strategies different from that of a classical camk2. The similarity with nearest homologues (members of camk2 subfamily) is confined to the catalytic domain.

#### ENSP00000363115 and ENSP00000229470

These two kinases are classified as ptk1 (src subfamily) in the current literature, but according to LMS scores, these two proteins are closer to subfamily of ptk8. ENSP00000363115 corresponds to Swissprot entry FGR_HUMAN, labeled as src Tyr kinase. There is experimental evidence at protein level for this entry [43] but its substrate specificity and mode of regulation are unknown. Its catalytic domain is tethered to SH3 and SH2 domains; similar domains are seen in close ptk8 members (ENSP00000348295 and ENSP00000357656). ENSP00000229470 also displays a SH2 domain, and is close to the same ptk8 members. It corresponds to a swissprot entry (Q5R3A8) which has no experimental characterization available. It should be noted that both these proteins have been assigned to ptk1 group with good confidence: 59% and 97% identity with catalytic domains of ptk1. However, they are both multi-domain proteins. Their domain organization places them close to the ptk8 subfamily, which is why we suggest a hybrid ptk1/ptk8 status. It is likely that the SH3 and SH2 domains tethered to the Tyr kinase domain facilitate protein-protein interactions while the kinase domain potentially elicit substrate specificity characteristic of ptk1 subfamily of tyrosine kinases. Existence of such kinases also raises the possibility of these kinases acting as a mediator of cross-talks between two signaling pathways.

#### ENSP00000354170

This kinase is traditionally classified as ptk3 (csk subfamily), with low similarity (31% identity with catalytic domains of csk subfamily). It corresponds to Swissprot entry Q59FL9, for which only transcript evidence is available. According to the present classification protocol, this kinase appears very close to many ptk15 members. Accessory domains immunoglobulins and I-set have been detected in this sequence, unlike other ptk3 but like ptk15 kinases. In addition, it has a few predicted trans-membrane segments. In view of these features, this kinase is likely to share the properties from ptk3 and ptk15 subfamilies.

#### ENSP00000343940

This sequence is classified as raf kinase with low similarity (33% identity with catalytic domain of raf). It corresponds to Swissprot entry TESK2_HUMAN, annotated as TKL Ser/Thr kinase. It has been characterized experimentally for its capacity to phosphorylate cofilin [44]. Based on LMS distance, it is very close to the category of “unclassified kinase” suggesting that the sequence outside the catalytic region is not typical of any of the known subfamilies. We thus suggest the status of hybrid raf/unclassified for this kinase, which is a mono-domain protein (catalytic domain encompass 277 out of 542 residues). This status implies that while this kinase might share some of the properties of raf kinase, it is unlikely to function as a typical raf kinase, with differentiation from classical raf kinases potentially mediated by the regions outside the catalytic domain.

#### Unclassified protein kinases

In addition to hybrid kinases, the LMS distances can suggest potential classification of kinases that do not fall in existing subfamilies. By examining the closest sequences for 83 unclassified kinases, we detected similarity with known subfamilies for 21 cases. In addition, 41 sequences show good similarity for other unclassified kinases and not pre-classified kinases. We suggest that these proteins could form one or several distinct novel subfamilies. The remaining 21 proteins display equivalent similarity to sequences from different subgroups, thus we cannot suggest any new membership. These results can be found in Table S4.

### Analysis of *Entamoeba histolytica* kinome

We used the set of protein kinases of E. histolytica stored in the KinG data base. Among these 307 sequences, 195 are assigned to a particular subfamily. The distribution of AUC values obtained after the iterative procedure suggests a threshold for hybrid detection equal to 0.84 (data not shown). AUC values are given in Table S5. As the experimental information about E. histolytica protein kinases is too sparse (only one reviewed Uniprot entry), we comment here on only a few cases.

One of the kinases, 304.m00063, is classified as agc1 based on its catalytic domain sequence. This sequence has an AUC value equal to 0.7 and has an unusual architecture, with a PH domain tethered to the catalytic domain. PH domains are usually seen in agc3 kinases. Interestingly, the closest neighbor of this sequence (4.m00671) is classified in the agc3 subfamily. A sequence classified in the pak subfamily, 157.m00090, has an AUC value equal to 0.7. This sequence has a predicted transmembrane domain, which is unusual for this subfamily. It is close to other sequences with transmembrane domains: 383.m00035, a raf hybrid kinase, and 232.m00085, an unclassified kinase.

### Conclusions

In their pioneering work, Hanks and Hunter presented the first classification of protein kinase subfamily [25]. They used phylogenetic analysis of catalytic domains to build classification trees, used as a basis for the classification in 55 distinct subfamilies. With similar objective, we have devised a classification strategy based on alignment-free measures and AUC computation. The alignment-free approach employed in this work enables convenient comparison of multi-domain kinases. Its fast computation allows its use on large data sets as well. Consideration of non-kinase regions in multi-domain kinases enables more robust classification and also enables recognition of hybrid kinases.

## Materials and Methods

### Data set

#### KinG Database

The KinG database (http://hodgkin.mbu.iisc.ernet.in/~king/) currently stores the analysis made on the kinomes of 488 organisms: 54 eukaryotes, 49 archaebacteria, 259 eubacteria, and 126 viruses. The procedure of detection and analysis of protein kinases have been described in several previous studies and is hence only briefly summarized here [8], [14]–[16], [20], [22]. Putative protein kinases are detected using a combination of profile search methods. Sequences that lack crucial functional residues in the catalytic domain are discarded. Putative functional kinases are assigned into one of the 55 Hanks and Hunter subfamilies (description in Table S6) using the following heuristic. Each sequence is matched against a database of multiple PSSM [45] that describes the catalytic domain for each of the 55 subfamilies, using rps-blast [46]. Hits with less than 30% sequence identity or less than 70% coverage of the query sequence are discarded to ensure that the putative protein kinase has a full catalytic domain. Each sequence is finally assigned to the subfamily which shows the best e-value. The domain annotation is done using HMMER [47] (http://hmmer.wustl.edu/), with e-value  = 0.01. In this study, subfamily assignment provided by KinG is used as a representative of Hanks and Hunter classification scheme.

#### Human kinome

We considered an updated version of human kinome analyzed in our group [48], following our first kinome annotation [8]. The full human kinome consists of over 550 proteins, with many of them assigned to one of the 55 Hanks and Hunter subfamilies using the method described above. Sequences and KinG annotations are available in Text S2.

#### Validation data set

In order to assess the validity of our approach, we consider a subset of the human kinome denoted as the validation data set. In order to eliminate any risk of misclassification that can occur when sequences exhibit very low sequence identity with the reference Hanks and Hunter profiles, we retain only sequences that are annotated in Swissprot. For the sequences present in Swissprot, the biological annotation is examined and they are kept only if the Swissprot annotation is in agreement with our KinG classification. It results in a data set of 212 sequences. The annotation provided by Swissprot can confirm the broad group of the kinase (e.g., cmgc), but not the exact subfamily (e.g. cmgc5). The 212 kinases are thus classified only in terms of broad groups, in this case, 17 groups. A total of 65 distinct domain architectures are observed. The composition of the validation data set in terms of broad groups and domain architecture is given in Table 1. Some families are well represented, like ptk with 51 members, whereas for other groups, we only have a few representative sequences, like translationk and wee1 for which there are only two members. The extreme case is the polo group, with only one sequence. The number of domains in one protein ranges from 1 to 11 (protein ENSP00000330161, a plantkr kinase in which the catalytic domain is followed by 10 ankyrin repeats). The number of different domains in one protein ranges from 1 to 6 (ENSP00000355237 and ENSP00000355727, two agc kinases where the catalytic domain is followed by a protein kinase C terminal domain, a DMPK coiled coil domain, a C1 domain, a pleckstrin homology domain and a CNH domain; ENSP00000261833, an agc kinase where DMPK coil is replaced by ATG16). Except the basic architecture made of a single catalytic domain (indicated by a * in the table), which is seen in 13 groups, no architecture is shared by different groups. Sequence ids are available in Table S7.

#### A protozoan kinome: *Entamoeba histolytica*

We consider the kinome of *Entamoeba histolytica*, the etiological agent of amoebiasis. The protein kinases encoded in the genome of E. histolytica have been analyzed in our group [22] and are available trough the KinG database. It is a set of 307 sequences, 195 of them being classified in one of the Hanks and Hunter subfamilies. Sequences and annotations are available in Text S3.

### Distances between sequences

#### Alignment-based distances

The global alignment between two sequences is computed by the Needleman-Wunsch algorithm implemented in the EMBOSS package [49], with following parameters: gap opening penalty = 10, gap extension penalty = 0.5, scoring matrix = BLOSUM62. From the global alignment, we derived two normalized distances. The first one, denoted as *identity distance* is simply defined by:
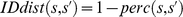
(1)where 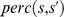 denotes the percentage of sequence identity between 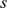 and 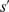, given by the number of identical residues divided by the alignment length. This quantity is relevant only to compare sequences with similar length and domain organization; it was thus applied to catalytic domains only.

The second distance, denoted as *BLOSUM distance*, is defined by:
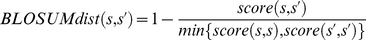
(2)where 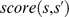 denotes the score of the alignment between 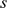 and 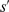, obtained using the Needleman-Wunsch algorithm, and 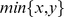 the minimum taken over 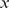 and 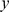. We also tested this last distance using gap opening penalty = 0 and gap extension penalty = 0, but it gave poor cluster quality.

#### Alignment-free distance based on the local matching score (LMS)

**Figure 5:**
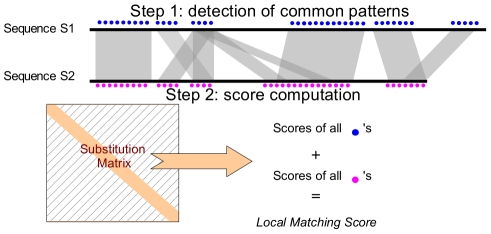
Computation of Local Matching Score (LMS) between two sequences without alignment.

We introduce a new similarity measure, called LMS for *Local Matching Score*, to assess local similarity between sequences without the use of sequence alignment. The principle is outlined in Figure 5. It is in essence similar to what is proposed in the CLUSS software [40], but we essentially introduced modifications in the normalization scheme.

The principle is to detect identical short patterns of length 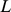 that are common to two sequences 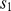 and 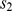. This can be achieved without alignment, by scanning 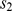 for all patterns present in 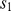. Once common patterns have been identified, the corresponding residues are scored using a substitution matrix (precisely, the diagonal scores of the matrix).

The local matching score (LMS) between sequences 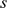 and 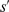 is thus given by:
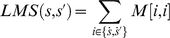
(3)where 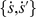 denotes the set of amino acids in 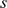 and 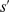 that are embedded in common patterns, and 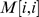 is the corresponding substitution score taken in a substitution matrix. We tested various minimum length from 2 to 6 and four types of substitution matrix: BLOSUM62, PAM250, PAM100 and the identity matrix. Scores are normalized to distances in the range [0,1] using the following formula:
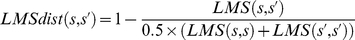
(4)


For LMS computation, the minimal length of common patterns is set to 5 residues. Scores are computed using the BLOSUM62 matrix, which gave slightly better performance than PAM250 (data not shown).

### Assessment of distances for sequence classification

#### Hierarchical clustering

We assess the ability of distances between sequences to form homogeneous clusters in a validation data set, as illustrated on Figure 3B. We used the agglomerative hierarchical clustering approach, under the R statistical environment [50], to generate a classification tree. The general principle of agglomerative hierarchical clustering is to iteratively merge the closest clusters, until we get one cluster containing all the data. Initially, each object to be classified is first assigned to its own cluster. One starts with a 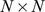 distance matrix describing the dissimilarities between the 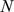 objects. After a new cluster has been formed, one has to compute the distance between this new cluster and all the others. Several linkage procedures are available to compute the distance between clusters. We used the Ward's linkage: the distance between two clusters is the increase of variance observed after merging the two clusters.

#### Measuring cluster quality

In the present case, all sequences are protein kinases, classified in different subfamilies according to Hanks and Hunter classification. In an ideal classification: (i) all members of a given sub-family should be grouped in the same cluster and (ii) each cluster should contain only one sub-family. These two properties are measured respectively by the *concentration* and the *purity* of the clusters. Let us denote by 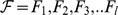 the set of subfamilies, and by 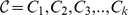 the set of clusters. The concentration of a sub-family 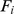 in a cluster 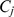 is the fraction of sequences in 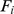 that belong to cluster 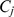; and the purity of cluster 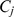, related to sub-family 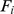 is the fraction of cluster 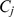 that belongs to sub-family 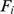. Clearly, as the number of clusters increases, purity will increase and concentration will decrease. For this reason, it is desirable to have a global measure that should be maximized. We used the Biological Homogeneity Index (BHI), introduced by Datta and Datta [51] to overcome the limitations of purity and concentration measures. It is defined by:
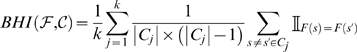
(5)where 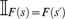 is equal to 1 if sequences 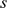 and 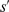 belong to the same subfamily and 0 otherwise. The BHI index is a measure of cluster homogeneity with respect to some biological classes. It is in the range [0,1], with larger values corresponding to more homogeneous clusters. Note that the splitting of one 100% pure cluster into two sub-clusters will let the BHI value unchanged.

### Detection of outliers in a set of pre-classified sequences

**Figure 6:**
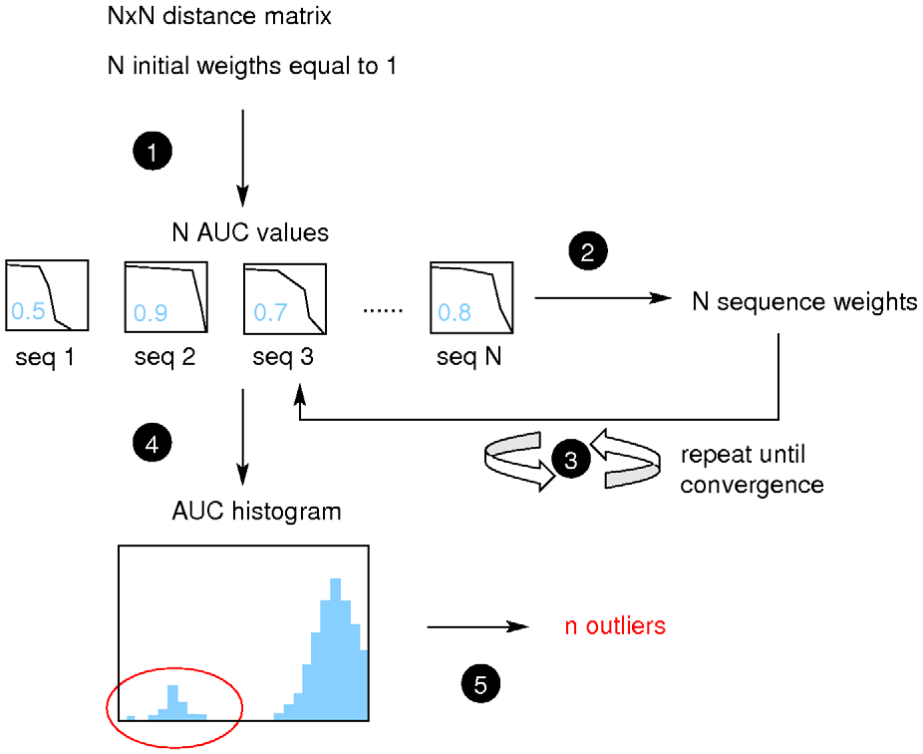
Detection of outliers in a pre-classified data set. 1: the distance matrix and initial weights are used to compute AUC values for each sequence using equation 6; 2: sequences weights are updated using equation 7; 3: the procedure is iterated until convergence; 4: the final AUC values are used to compute a histogram; 5: the histogram shape is used to detect outliers.

**Figure 7:**
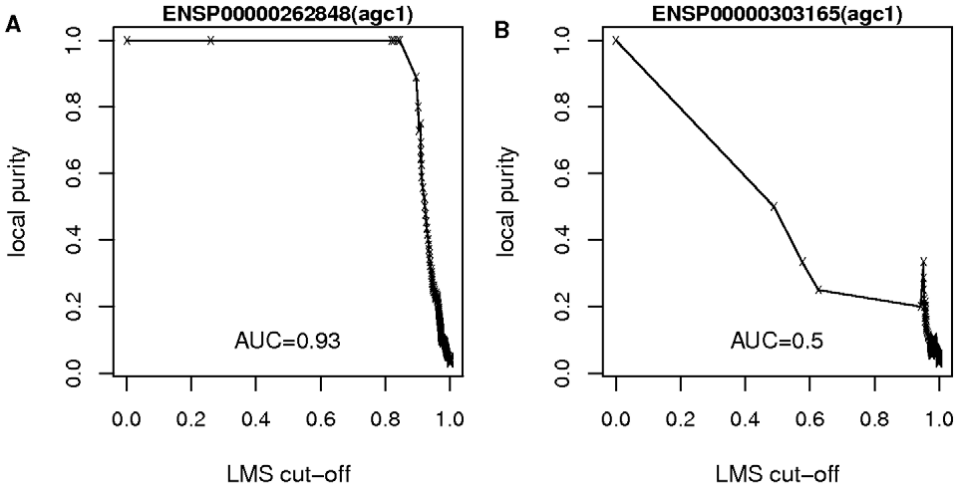
Examples of classification curves. A: a putatively well classified sequence, B: a putatively misclassified sequence. AUC denotes the area under the curve.

Starting from a set of protein kinases already classified in terms of Hanks and Hunter subfamilies based only on the similarity of their catalytic domains, our goal is to use full-length distances to detect the sequences that are outliers in the initial classification. We use an iterative procedure, as illustrated in Figure 6. The idea is to set a sequence as the reference point, and consider its close neighbors: if close neighbors are classified in subfamilies other than the reference, the reference sequence is an outlier, potentially misclassified. We start initially with a distance matrix and a set of sequence weights 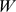, all equal to 1. For each sequence 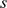, we plot the local purity 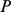 *versus* distance cut-off 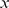, and compute the Area Under the Curve (AUC) as follows:
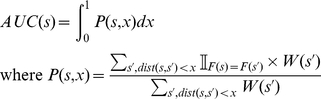
(6)where 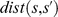 denotes the LMS distance between s and s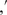 and 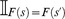 is equal to 1 if sequences 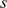 and 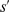 belong to the same subfamily and 0 otherwise. The shape of the curve 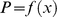 is an indicator of the similarity of a sequence with members of the same subfamily and others, as illustrated on Figure 7. In the case of a correct classification (Figure 7a), the nearest neighbors of a reference sequence are from the same subfamily, and sequences from different subfamilies are more distant. It results in a high local purity at low distance, a decrease of purity at high distance and an AUC value close to 1. On the contrary, if a sequence is misclassified (Figure 7b), the local purity is low even at low distance, resulting in low AUC value. This approach has an obvious limitation: the presence of misclassified sequences at short distance of a reference sequence might distort its purity curve and alter its AUC. To circumvent this limitation, we iterate the procedure by updating the sequence weights at each iteration as follows:
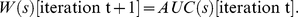
(7)


The procedure is iterated until the AUC values remain constant. The putatively misclassified sequences are detected using the value of AUC: sequences with an AUC lower than a chosen cut-off are proposed as hybrid kinases. The final AUC cut-off is chosen according to the distribution of AUC: for both human and *E. histolytica* sequences, we observed a clear bimodal shape for the LMS distributions. Such bimodal distributions suggest that two types of sequences are present in the data set: well-classified sequences with high AUC values, and divergent, putatively misclassified sequences, with low AUC values. The cut-off was then naturally chosen at the limit between the two modes. BLOSUM distances did not produced bimodal AUC distributions.

## Supporting Information

Text S1 Preliminary results obtained using domain-based distance measures.
(0.24 MB PDF)

Text S2 Fasta sequences and KinG information on human kinome.
(0.60 MB TXT)

Text S3 Fasta sequences and KinG information on *E. histolytica* kinome.
(0.27 MB TXT)

Table S1 Summary information on hybrid kinases in human kinome. id: percentage of identity with the best matching catalytic domain profile, cov: percentage of coverage with the best matching catalytic domain profile, closest sequences: subfamily classification of the closest sequences based on LMS distance, dist: lowest LMS distance in the whole data set.
(0.02 MB XLS)

Table S2 Detailed information on hybrid kinases in human kinome. For each hybrid kinase, we report the domain architecture (specifying the Pfam domain id, position in sequence and HMMer e-value), and the domain architectures of the five closest sequences. Sequences indicated by * are hybrids that appear close to other hybrids.
(0.04 MB XLS)

Table S3 AUC values of human kinases.
(0.05 MB XLS)

Table S4 Detailed results about unclassified human kinases.
(0.02 MB XLS)

Table S5 AUC values on *E. histolytica* kinases.
(0.04 MB XLS)

Table S6 Definition of the 55 subfamilies of the Hanks and Hunter classification.
(0.01 MB XLS)

Table S7 List of ids for the 212 proteins of the validation data set.
(0.02 MB XLS)
