# Supplementary material for: Classification of Protein Kinases on the Basis of Both Kinase and Non-Kinase Regions
Source: PLoS One. 2010 Sep 15;5(9):e12460. doi: 10.1371/journal.pone.0012460 (PMC2939887; doi:10.1371/journal.pone.0012460)
Supplement: Text S1 — Preliminary results obtained using domain-based distance measures. (0.24 MB PDF) [file pone.0012460.s001.pdf]

Definition of architecture-based similarity measures

The similarity between two domain architectures can be assessed using various measures, summarized in [**?**]. Measures tested in this study are listed below.

The number of common domains has been used to detect protein of similar architectures in CDART (Conserved Domain Architecture Retrieval Tool) [**?**]. It is obtained by simply counting identical domain pairs in two architectures. For example, the number of common domains between architectures 'ABC' and 'CBD' is equal to 2, and 3 between 'ABBA' and 'BABC'. This distance is denoted n_{common}.

The Jacquard similarity index is given by:

$$J(p_1, p_2) = \frac{n_{common}}{n_1 + n_2 - n_{common}}$$

where n_1 and n_2 are respectively the number of domains in proteins p_1 and p_2. This index is based on the assumption that long proteins are more likely to carry similar domains than short ones, it thus introduces a length correction to the previous measure.

All domains are considered equally when computing such scores. Another approach consists in attributing some weights to the domains, in order to give more importance to informative domains than uninformative ones (weighting schemes are detailed in the next subsection). Then, some similarity scores have been proposed that rely on domain weights.

A simple similarity score based on weights is given by:

$$S(p_1, p_2) = \sum_i w(d_i, p1) w(d_i, p2).$$

where p_1 and p_2 denote protein architectures. The sum is taken over all domains seen in p_1 and p_2 and domains weights can be computed using several schemes, described in the next subsection. This similarity score is denoted SimScore.

The cosine similarity measure is given by:

$$C(p_1, p_2) = \frac{\sum_i w(d_i, p_1) w(d_i, p_2)}{\sqrt{\sum_i w(d_i, p_1)^2 \sum_i w(d_i, p_2)^2}}.$$

It is the cosine of the angle formed by the two protein vectors when proteins are translated into weight vectors. The cosine similarity measure is similar to the simple similarity measure, but it is normalized by the product of vector norms, a number related to the length of the proteins.

It should be noted that these last two scores can also be employed in the absence of weighting scheme, by using un-normalized domain occurrence in place of weights.

The similarity introduced by Lin et al [?] is a weighted combination of three terms: (i) the Jacquard similarity index, (ii) a term derived from the Goodman-Kruskal γ index, and (iii) a domain duplication term. The Goodman-Kruskal γ index for two protein p_1 and p_2 is given by:

$$\gamma(p_1, p_2) = \frac{N^S_{p_1 p_2} + N^r_{p_1 p_2}}{N^S_{p_1 p_2} - N^r_{p_1 p_2}}$$

where $N^S_{p_1 p_2}$ is the number of domain pairs found in the same order in proteins p_1 and p_2, and $N^r_{p_1 p_2}$ is the number of domain pairs found seen in the reverse order in proteins p_1 and p_2. The γ index is normalized to fit the range [0,1]: $G(p_1, p_1) = \frac{1-\gamma(p_1,p_2)}{2}$. The domain duplication similarity is given by:

$$D(p_1, p_2) = \exp(-\sum_i \frac{|N(i, p_1) - N(i, p_2)|}{S})$$

where $N(i, p_1)$ denotes the number of domain i in protein p_1. The sum is taken over all the domains seen in p_1 and p_2, and S is given by:

$$S = \sum_i max(N(i, p_1), N(i_p 2)).$$

Finally, the Lin similarity is given by:

$$Lin(p_1, p_1) = 0.36J(p_1, p_2) + 0.01G(p_1, p_2) + 0.63D(p_1, p_2).$$

The distance introduced by Björklund et al [?] is defined by the number of unmatched domains after a global alignment. The global alignment is obtained using the Needleman-Wunsch algorithm implemented in EMBOSS package [?], with the following parameters: match is is given score 1, mismatch is given score 0, gap open penalty is 0.01, gap extension penalty is 0.001. These are the parameter values proposed in [?].

Weighting schemes

Domain weights can be used to correct bias induced by the uneven repartition of domains. We tested the weighted schemes introduced by Song et al [?].

Distinct Partner (dp) weights allow to give lower weight to domains that are seen tethered with a variety of different domains. The idea is to penalize ubiquitous domains. For a given domain d, it is given by:

$$W_{dp}(d) = \frac{1}{N_{dp}(d)}$$

where N_{dp} is the number of distinct partners that are seen co-occurring with domain d in the full data set.

Inverse document frequency (idf) scheme gives low weight to domains occurring in many proteins and, inversely, high weight to domains occurring in few proteins only. It is given by:

$$W_{idf}(d) = \log_2 \frac{N_{prot}}{N_{prot}|d \in prot}$$

where N_{prot} is the total number of proteins in the data set and the denominator is the number of proteins containing d.

Tf-idf weights (tf-idf) are obtained by combining idf weights with *text-frequency* weights (tf). Tf weights give high weight to domain that are frequent in a given protein. They are defined by:

$$W_{tf}(d, p) = \frac{N(d, p)}{N(., p)}$$

where $N(d, p)$ denotes the number of domain d in the protein p and $N(., p)$, the total number of domains in protein p. The tf-idf weights are defined by:

$$W_{tf-idf}(d, p) = W_{tf}(d, p) \times W_{idf}(d).$$

Domain copy correction

Architecture-based distances can be computed with or without *domain copy correction*: for example, architectures 'ABBA' and 'BABC' share three domain copies (one A and two B), but only two domain types (A and B).

Preliminary results on Hanks and Hunter and human kinome data sets

Alignment-based classification

Hanks and Hunter data set: the evolution of BHI, measuring cluster homogeneity is shown on Figure 1. The maximum BHI is obtained for 24 clusters and is equal to 0.724.

Human kinome data set: Only the catalytic sites of human kinases are considered. The evolution of BHI is shown on Figure 1. The maximum BHI is obtained for 99 clusters and is equal to 0.87.

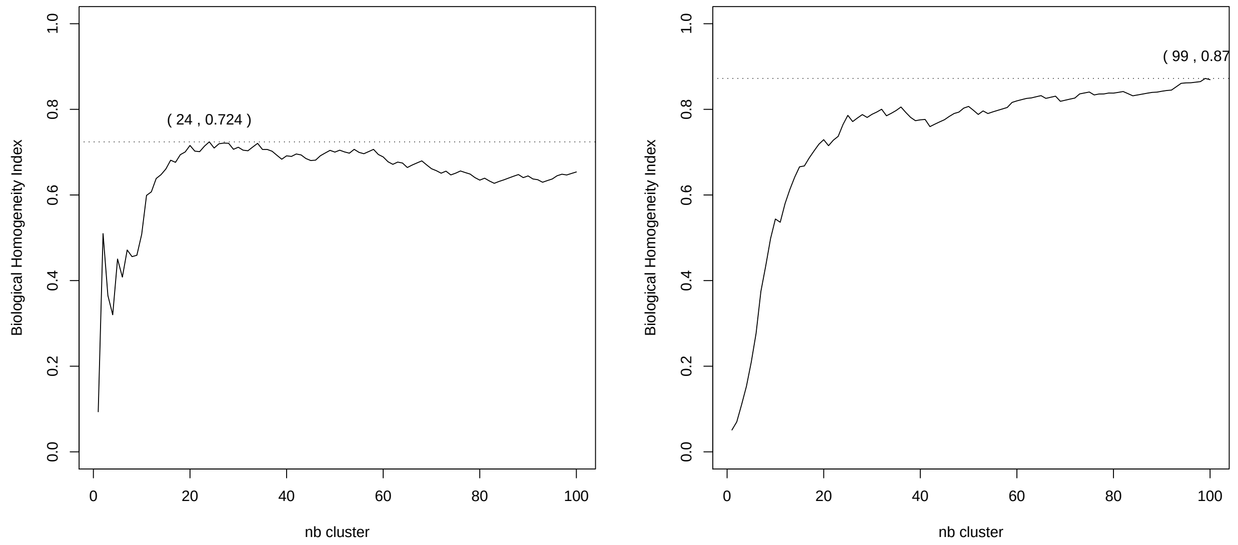

Figure 1: Evolution of BHI according to the number of clusters using alignment based similarity. Left part: clustering of Hanks and Hunter data, right part: clustering of human kinome data (catalytic domains only).

Architecture-based classification

Human kinome data set: We compute architecture based similarity measures using the four measures described in section (n_{common}, Jacquard, SimScore, cosine, Lin similarity and Bjrklund distance), combined with the three weighting schemes (dp, idf, tf-idf), with and without domain copy correction.

The evolution of BHI, purity and concentration is shown on Figure 2.

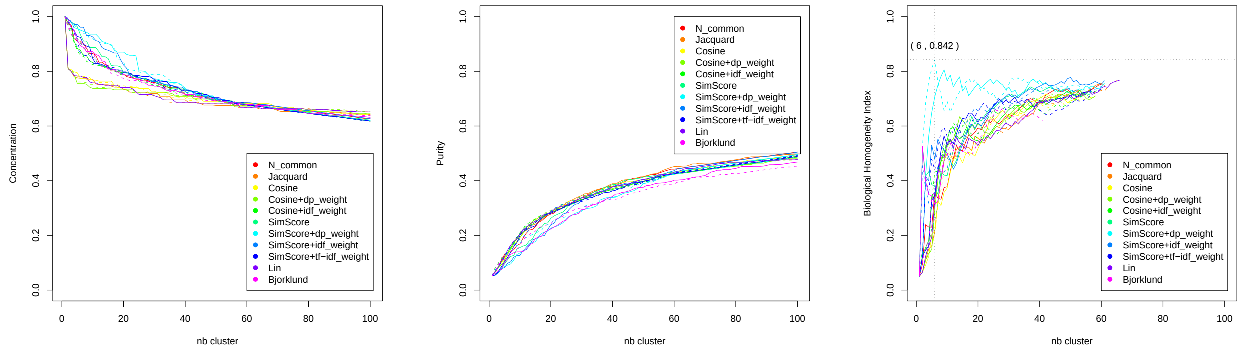

Figure 2: Evolution of cluster quality measures according to the number of clusters using architecture-based similarity measures, in the human kinome set. Plain lines: no domain copy correction, dashed lines: domain copy correction is used.

It can be seen on Figure 2, that the cluster homogeneity, measured by the BHI, display roughly similar evolution, whatever measure or weighting scheme is used. The only notable exception is the SimScore measure used with dp weights: when domain copy correction is applied, is generates the partition with the maximum BHI: 0.842, for 6 clusters. The evolution of concentration an purity measures, indicates that the SimScore measures, used in combination with dp and idf weighting scheme, allows to significantly retain high concentration for number of clusters lower than 30. In the range [40-60] clusters,the best BHI (0.767) is obtained for SimScore with idf weights, at 52 clusters, see 2.

Combination of alignment-based and architecture-based similarity

The pairwise identity percentage of catalytic domains and the SimScore with idf weights are combined into a single measure. Both similarity tables are converted into distance measures and SimScores are re-scaled to be in the range [0-100]. The two distances are then combined using the following formula:

$$D = (1 - \alpha)D_{pairwise} + \alpha D_{archi},$$

where $D_{pairwise}$ denotes the distance derived from pairwise identity percentages and D_{archi} denotes the distance derived from SimScores+idf weights. When $\alpha = 0$, the clustering is based on catalytic domain sequence similarity, when $\alpha = 1$, the clustering is based on similarity between domain architectures.

The result is shown on Figure 3.

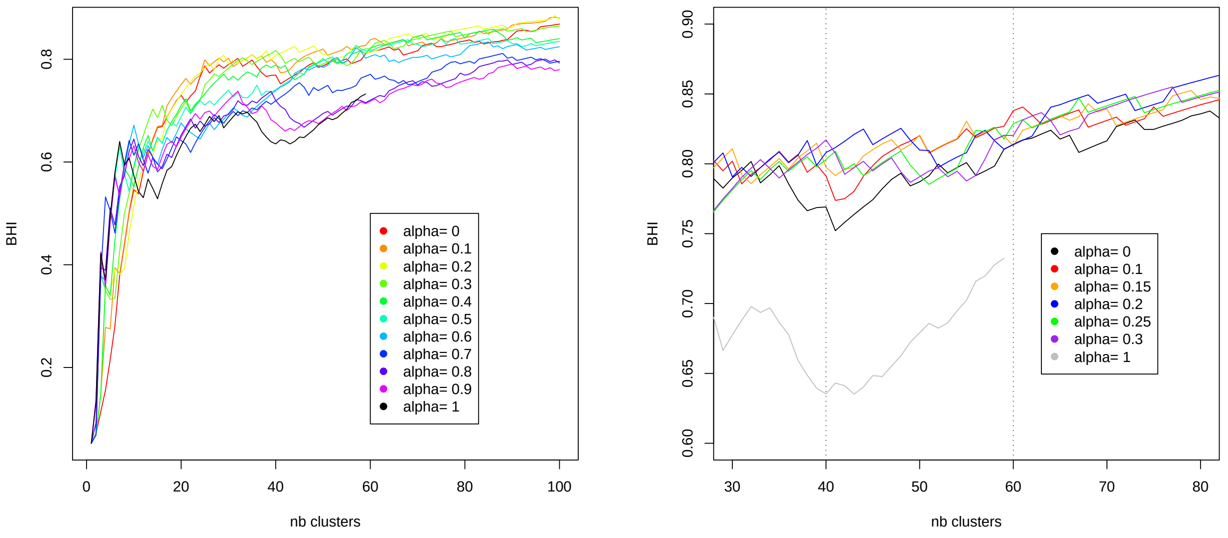

Figure 3: Evolution of BHI according to the number of clusters using a combination of alignment based and architecture based similarity measure. Left part: α range from 0 (purely alignment based) to 1 (purely architecture based). Right part: α range from 0 to 0.3.

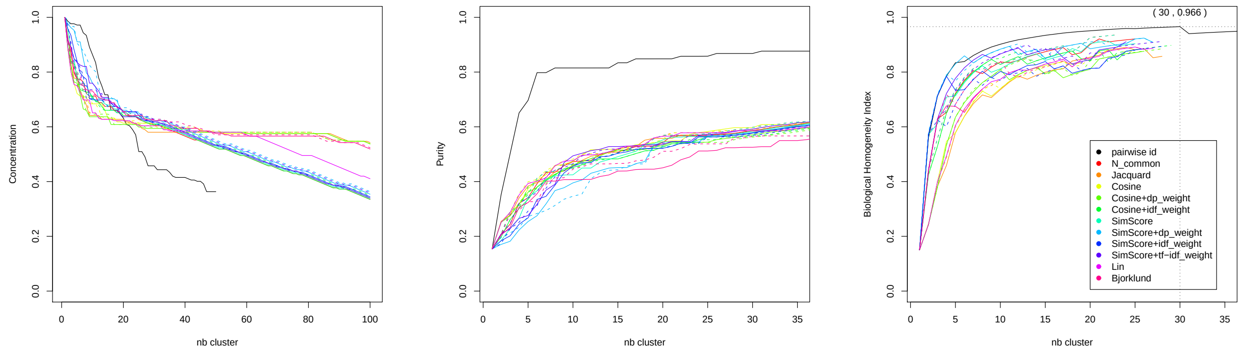

Figure 4: Evolution of cluster quality measures according to the number of clusters during the classification of the validation data set. Pairwise id (black line): classification based on sequence similarity between catalytic domains, others (color lines): classification based on the domain architectures, plain lines: no domain copy correction, dashed lines: domain copy correction is used.

It is interesting to note that although alignment based similarity ($\alpha = 0$) clearly generated more homogeneous clusters than architecture based similarity ($\alpha = 1$) , the combination of these two measures improves the BHI index compared to separate measures. A choice of α around 0.2 yields the highest BHI index in the range 40 to 60 clusters. This result indicates that a slight contribution of architecture based similarity (here, 20%) could help to generate homogeneous clusters.

Classification of the validation data set

Alignment-based classification

Cluster quality measures, i.e., BHI, concentration and purity, are reported in Figure 4. The maximum BHI, equal to 97% is obtained for 30 clusters. The examination of purity and concentration evolution shows that the concentration of each sub-family is high in the range 1-5 clusters and then rapidly decreases. It thus indicates that a small number of clusters achieves a correct sub-family separation (in terms of concentration), that is rapidly lost as the number of clusters increase.

To better understand the qualitative separation of the proteins during the clustering procedure, we display the hierarchical classification tree in Figure 5 (left part). As the validation data set is made of protein kinases with from different broad functional groups and unclassified kinases, we display the groups that are obtained by dividing the tree into 16 sub-trees using a constant depth cut-off. Three main remarks can be made from the tree shown on Figure 5: (i) proteins from the same group cluster well together, (ii) unclassified kinases cluster in various part of the tree, (iii) by cutting the tree at a given depth results in the splitting of some families (like camk, cmgc, agc), whereas some others are grouped together in a mixed cluster(e.g., nima, wee1, translationk, plantrk). 37 sequences are included in the mixed cluster.

Architecture-based classification

The evolution of BHI, concentration and purity is shown on Figure 4. All the similarity measures give the similar BHI profiles: increase up to 15 cluster, then decrease and increase again. The SimScore used with dp and tf-idf weights seems to give the best results in the rage [0-15]. Interestingly, the concentration profiles indicate that the SimScore with dp-weights provides highest concentration for a small number of clusters. The corresponding hierarchical clustering tree is shown on Figure 5 (right part). The domain architecture is a very crude information compared to sequence identity, which explains that the clustering achieved using this criterion is less satisfying. As with sequence identity, the splitting of the tree at a fixed cut-off results in some families being split (agc, ptk), a large cluster of 130 sequences gathers protein kinases from different groups, including the 100 protein kinases with basic architecture. This cluster also contains all the unclassified kinases. The misclassified sequences identified in the pairwise identity-based tree have been highlighted. As most of them have a basic architecture, they fall in that particular cluster and not with their relatives. However, proteins ENSP371067 and ENSP264818(ptk), which were misclassified in the first tree re now clustered with other ptk.

Combination of alignment-based and architecture-based similarity

We use a linear combination of architecture-based and sequence similarity:

$$D = (1 - \alpha)D_{pairwise} + \alpha D_{archi},$$

where $D_{pairwise}$ denotes the distance derived from pairwise identity percentages and D_{archi} denotes the distance derived from domain architecture comparison. We consider the domain architecture similarity computed using the SimScore with dp weights, without domain copy correction. The evolution of BHI with the number of clusters, for different values of α are displayed on figure 6.

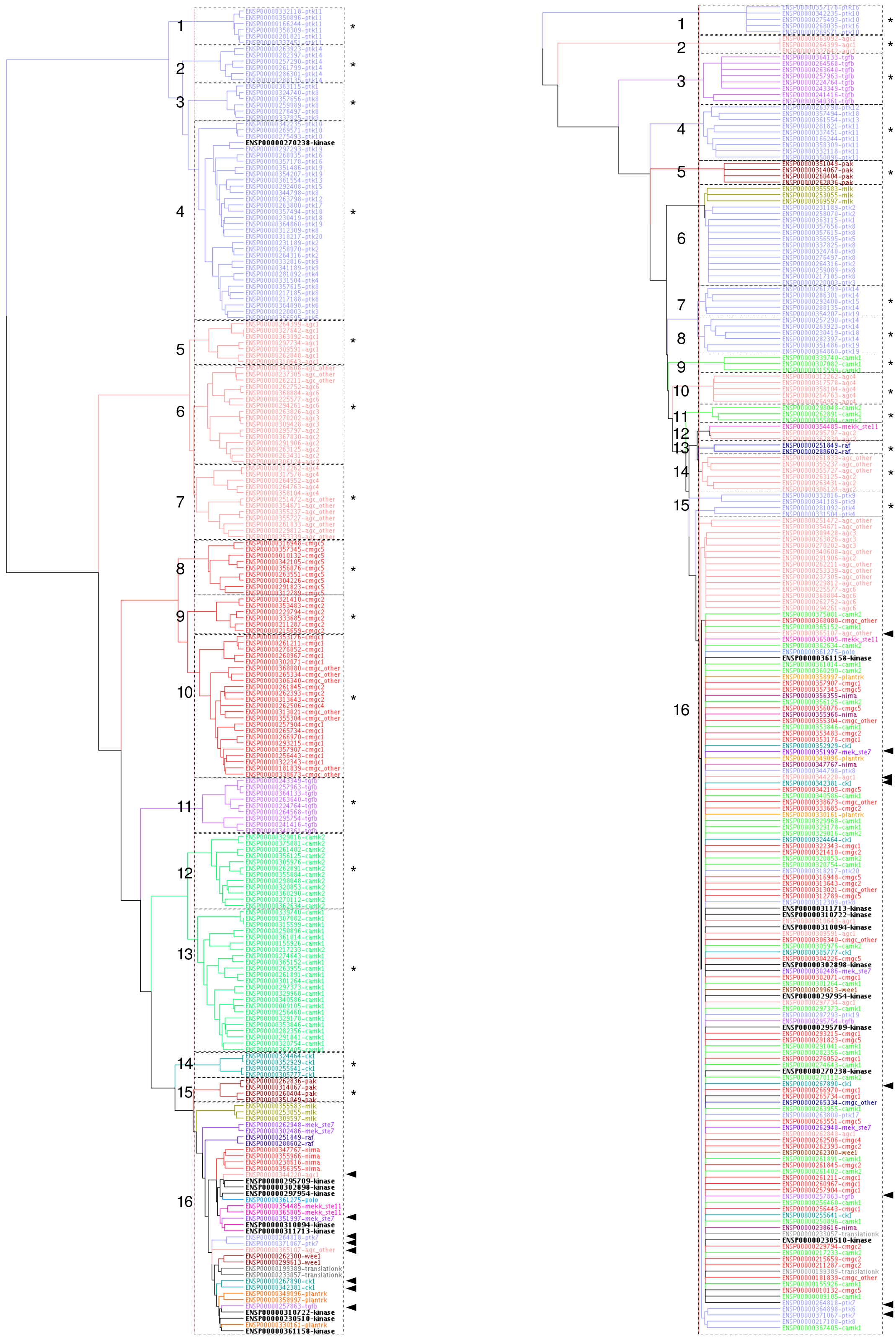

Figure 5: Hierarchical clustering tree of the validation data set. Different colors correspond to different functional annotations. Unclassified kinases are shown in bold face. Dashed rectangles indicate the clusters obtained by cutting the tree at a fixed depth. * indicates pure clusters. Left part: clustering based on sequence identity between catalytic domains. Black triangles indicate misclassified proteins.Right part: clustering based on the similarity between domain architectures, computed with the SimScore, dp weights, and without domain copy correction.

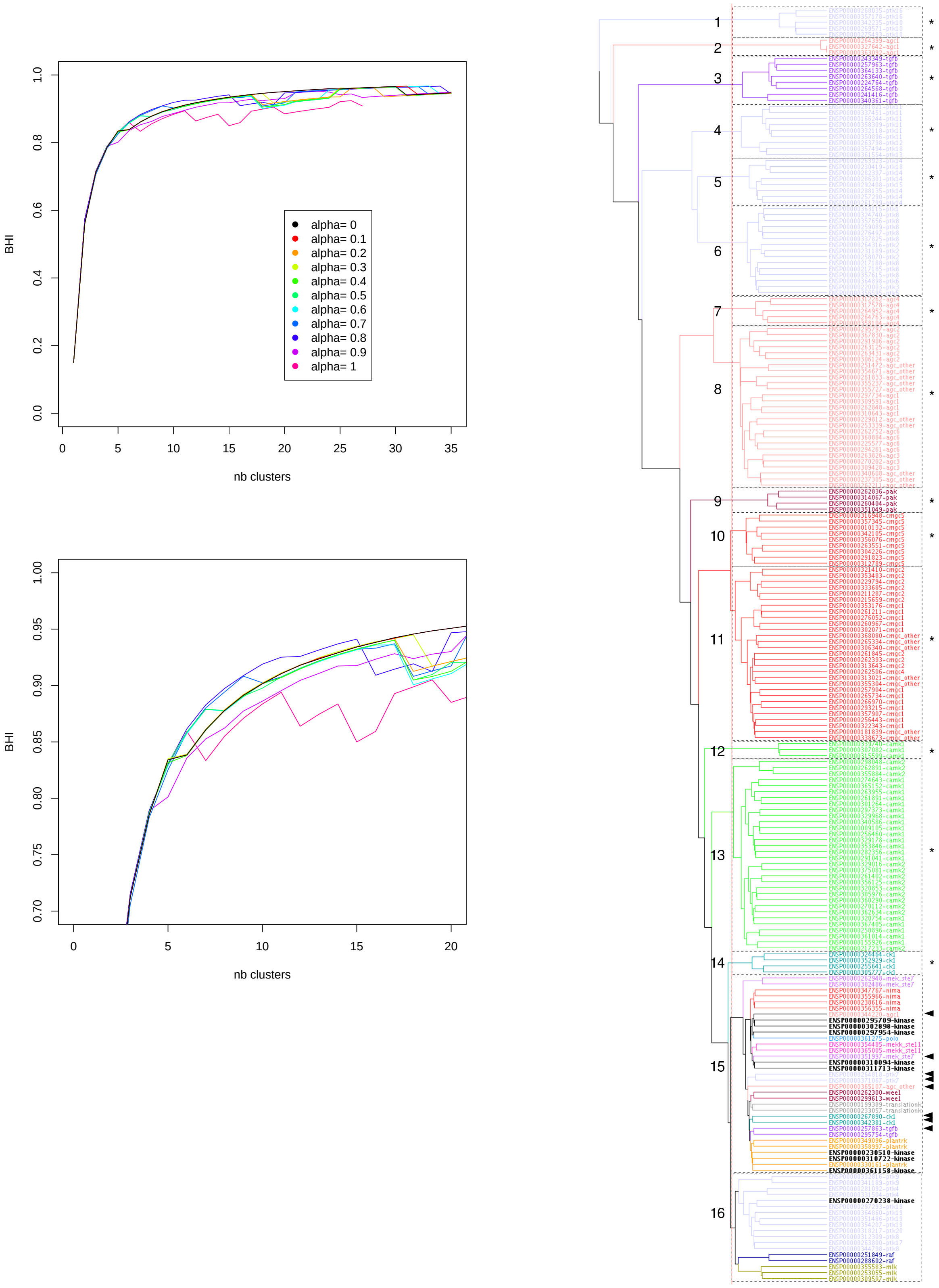

Figure 6: Hierarchical clustering of validation data set using a combination of sequence and architecture-based similarity. Left part: evolution of BHI with the number of clusters for different values of α. Right part: clustering tree obtained using $\alpha = 0.8$.
